# Navigating the challenges: ultrasound innovations in brain glioma surgery

**DOI:** 10.3389/fneur.2025.1553018

**Published:** 2025-10-01

**Authors:** Shayan Sadrinasab, Armin Aminiyan, Sadaf Saket, Fatemeh Khosravi, Nadia Pourmohammadi, Masoud Saadat Fakhr

**Affiliations:** ^1^Shahid Beheshti University, Tehran, Iran; ^2^Islamic Azad University System, Tehran, Iran; ^3^Shahid Beheshti University of Medical Sciences, Tehran, Iran; ^4^Islamic Azad University, Ramsar Branch, Iran; ^5^Shahroud University of Medical Sciences, Shahrood, Iran; ^6^Faculty of Medicine, Tehran Medical Sciences School Branch, Islamic Azad University, Tehran, Iran

**Keywords:** glioma surgery, intraoperative ultrasound, navigable ultrasound systems, brain tumor resection, functional ultrasound

## Abstract

Achieving maximal safe resection during glioma surgery while preserving neurological function remains a significant challenge. Intraoperative ultrasound (IOUS) offers real-time imaging that dynamically adapts to brain shift and surgical progression. This review highlights recent advances in IOUS, including established modalities such as contrast-enhanced and 3D ultrasound, and emerging innovations such as functional ultrasound (FUS), 4D volumetric imaging, artificial intelligence (AI)-assisted interpretation, and ultrasound-sensitive nanobubbles. These technologies aim to improve the identification of residual tumor, delineate infiltrative margins, and enable functional preservation. Integration with neuronavigation systems enhances accuracy, while new theranostic strategies suggest a future role for ultrasound in intraoperative therapy. Collectively, these developments position IOUS as a central component in the evolution of precision glioma surgery.

## 1 Introduction

Achieving maximal safe resection is the main goal of surgical treatment for malignant brain tumors. This approach aims to remove the tumor completely while sparing as much functional brain tissue as feasible. Patients with brain tumors, especially gliomas—the most prevalent malignant primary brain tumors—must have this procedure if their survival rates and quality of life are to be improved. The most aggressive and deadly subtype of brain tumors are glioblastomas, which make up 74.6% of malignant brain tumors and 24.7% of primary brain tumors overall (1). Because of their bad prognosis, glioblastomas continue to be a challenge to treat, even with newer protocols that include surgery, radiation, and temozolomide chemotherapy, such as the Stupp protocol. Accurately localizing tumor margins to achieve maximal excision without harming critical brain structures is a major difficulty in glioma surgery. Preoperative planning has been transformed by techniques such as frame-based and frameless stereotactic neuronavigation, which aid neurosurgeons in determining the best site for craniotomy. Intraoperative alterations, such as brain movement due to gravity, cerebrospinal fluid (CSF) drainage, and tissue deformation during tumor removal, are not taken into consideration by these systems since they rely on preoperative imaging (2). As the procedure continues, this restriction makes navigation systems far less accurate, necessitating alternatives that incorporate real-time photography. To overcome the shortcomings of preoperative navigation, intraoperative imaging has become an essential tool. Surgical teams now have access to real-time feedback thanks to techniques like intraoperative ultrasound (IOUS), intraoperative computed tomography (IOCT), and intraoperative magnetic resonance imaging (IOMR) (3). Among these, IOUS has become quite popular because of its affordability, mobility, and capacity to adjust to the ever-changing surgical industry. When it comes to real-time tumor viewing and resection, IOUS provides a more practical option to IOMR, which is costly and requires complicated preparations. The Dussik brothers first employed diagnostic ultrasound to find brain tumors in the 1930s; since then, the technology has found its way into neurosurgery. The use of A-mode ultrasonography to detect tumors had been around since the 1950s. Nevertheless, the use of IOUS as a useful tool for directing brain tumor resections did not become widespread until the 1980s, when real-time gray-scale B-mode imaging became widely available. Integrating intraoperative ultrasound (IOUS) into current neurosurgery procedures was made possible by ground-breaking research in the 1990s that demonstrated its efficacy in localizing tumor margins and easing gross total excision (4). Glioma surgery cannot be performed without IOUS because to its numerous benefits. Continuous monitoring of the surgical field is made possible by its real-time imaging capacity, which provides important information as the surgery progresses. This aids surgeons in properly resecting tumors by allowing them to account for brain shift and other intraoperative alterations. Further advantages of IOUS over IOMR or IOCT are its portability, user-friendliness, and low cost. Although it does add some time to the surgical process, it provides accurate, real-time input on the shape and borders of tumors (5). In order to successfully differentiate tumor tissue, IOUS depends on the hyperechogenic appearance of gliomas in comparison to the surrounding brain parenchyma. Expertise in interpreting ultrasound pictures is required, though, because things like tumor calcifications or blood might change its echogenicity. To achieve maximum safe resection, real-time imaging with IOUS is required for critical steps like designing the dura opening, focusing the corticectomy, and determining tumor boundaries (6). The evolution of ultrasound technology has made IOUS a powerful tool for multiparametric imaging. Its use in glioma surgery has been improved by innovations including contrast-enhanced ultrasound, sophisticated probes, and fusion imaging, which combines preoperative MRI/CT scans with IOUS. By superimposing real-time IOUS data onto preoperative images, fusion imaging overcomes the difficulty of anatomical orientation and contrast-enhanced ultrasound increases the sensitivity and specificity of tumor visualization (7). We must confront the limitations of IOUS notwithstanding its advantages. Accurate interpretation of IOUS images is highly dependent on the operator's training and expertise. Less experienced users may also find it tough to interpret images from IOUS because it does not adhere to typical anatomical planes like MRI or CT. And because it can't go inside the skull, IOUS is only useful for imaging after a craniotomy. These restrictions highlight the need for specialized education and the incorporation of supplementary imaging methods to enhance the efficacy of IOUS.

The absence of uniform training for neurosurgeons is a major obstacle to the broad use of intraoperative ultrasound guidance during glioma surgery. Knowing the ins and outs of the procedure and being well-versed in the anatomical details shown on screen are prerequisites for accurately interpreting IOUS images. To overcome this difficulty and enable more neurosurgeons to harness its benefits in clinical practice, systematic training programs and the incorporation of IOUS into neurosurgical curricula are needed (8). In addition to its remarkable success in glioma surgery, IOUS has found use in several fields of neurosurgery, including the assessment of cerebral blood flow using transcranial Doppler and the removal of various intracranial tumors. Because of its adaptability, intraoperative ultrasound (IOUS) has the makings of a gold standard in neurosurgery for both diagnostic and intraoperative purposes. Thorough assessments of IOUS's function in glioma surgery are necessary because of the technology's rising profile and quick development. The purpose of this review is to examine recent works on IOUS and to draw attention to its merits, shortcomings, and new developments (9, 10). We aim to shed light on the revolutionary potential of IOUS and propose techniques for making the most of it to achieve the safest possible glioma resection by combining data from current research.

## 2 Technological background

### 2.1 Fundamentals of ultrasound imaging

**Figure 1:**
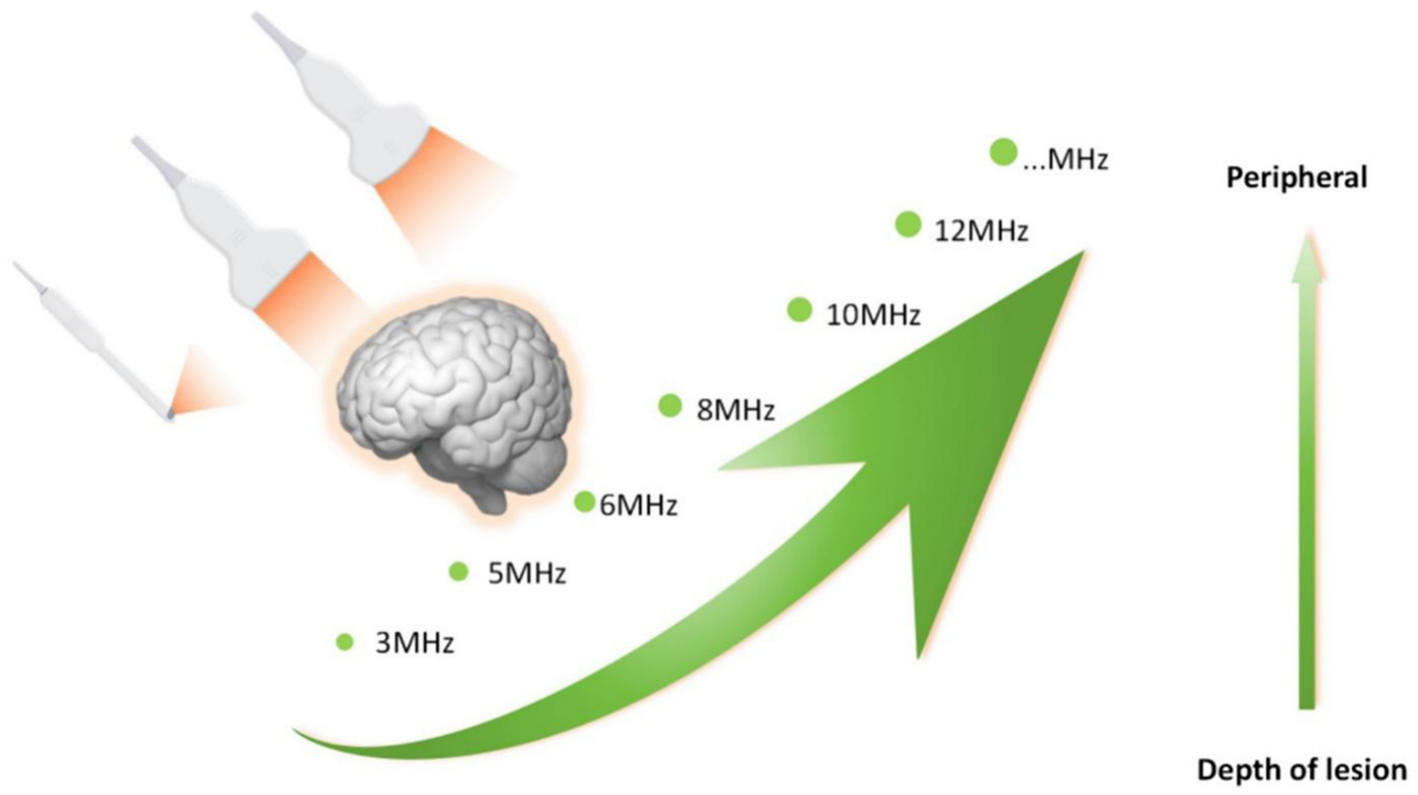
Various probes with matching field depth for real-time imaging (12).

In many ranches of surgery, ultrasound imaging has completely altered intraoperative procedures. Its applications include the detection of liver tumors, the resection of pancreatic cancer, the characterization of breast tumors, and laparoscopic procedures. Use of intraoperative ultrasound guidance (IOUS) has several applications in neurosurgery, including tumor localization, surgical planning, and evaluation of resection extent (EOR). Achieving maximum safe tumor excision relies on its real-time imaging capability, which is essential for intraoperative changes (11) (Figure 1).

There are special considerations while using IOUS in neurosurgery. The dexterity of the probe is generally limited by the size of the craniotomy, and efficient probe contact might be disrupted by resection voids that are generated during surgery. Because of these limitations, neurosurgical procedures require specialized probes that cannot be used in traditional surgical procedures, such as those intended for use in the abdomen or in pediatrics. In order to have accurate imaging during brain tumor procedures, it is essential to overcome these restrictions (13, 14). Sterilization techniques for ultrasonic probes must be strong for IOUS to be integrated into sterile surgical settings. There are three main methods that are commonly used for sterilization:

Sterilization with ethylene oxide gas is effective yet labor-intensive (16–24 h) and dangerously hot, potentially damaging delicate machinery. While immersing in glutaraldehyde shortens the turnaround time (approximately 4 h) and prevents heat exposure, it is not without risk owing to its irritating and allergic characteristics. Vapor-Gas of Hydrogen Peroxide Plasma: a more contemporary method that is compatible with many probe designs, it offers fast processing (2–3 h) without contact dangers. The sterilizing process that is used is contingent upon the type of probe and the preferences of the institution (13, 15). In order to get clear images during surgery, the probe must be properly coupled to the location. When the probes are placed on the dura or exposed brain tissue, sterile gel or saline can be used to improve acoustic coupling. Excessive pressure can deform the brain and reduce imaging accuracy, thus handling with care is crucial. Another way to reduce artifacts and make larger probes more accessible is to fill resection voids with saline. With IOUS, the tumor boundaries may be seen in great detail before resection, which helps with surgical trajectory planning. Improved imaging within resection areas is achieved during the process with smaller probes or saline-filled cavities (16). This allows for effective localization of residual tumor tissue. Research has shown that IOUS is useful for both decreasing residual volumes and attaining gross total resection (GTR). The use of IOUS has been greatly improved by recent innovations. Tumors can be better defined with contrast-enhanced ultrasonography, and methods like radiomics-based image processing provide accurate characterization and prognostic assessments. Furthermore, a hybrid imaging environment can be created using fusion imaging, which overlays preoperative MRI data with real-time ultrasonography. This helps to mitigate the effects of brain shift and aids in surgical decision-making (7, 17). The proficiency of the operator is crucial for the effective utilization of IOUS. It is essential that neurosurgeons participate in training programs that teach them to evaluate pictures in planes that are not typically used in the field. By providing a framework for structured IOUS training, institutions are able to increase adoption rates and decrease learning curves, which in turn improves clinical outcomes (18). While IOUS provides adaptability and real-time imaging, it does have several drawbacks, such as the need for an operator, difficulties in interpreting images, and the fact that imaging with IOUS requires physical touch. These obstacles should be surmounted by innovations including more compact and high-resolution probes, enhanced imaging techniques, and the incorporation of AI. Using IOUS is becoming more common for treatments involving juvenile brain tumors, cerebral vascular abnormalities, and glioma surgery. In a number of neurosurgical contexts, it has proven to be useful for obtaining localization with high precision and with little difficulties. As an innovative imaging technique, IOUS has the potential to revolutionize neurosurgery. A vital part of contemporary neurosurgery practice, it can give real-time information, adjust to surgical dynamics, and is cost-effective. It will become even more crucial in attaining the safest possible tumor resections as imaging technology, operator training, and probe design continue to progress (19, 20).

### 2.2 Key ultrasound modalities

#### 2.2.1 Two-dimensional ultrasound

Utilizing sound waves at frequencies ranging from 1 to 20 MHz, two-dimensional ultrasound (2D US) provides real-time, planar images of inside structures; it is a fundamental tool for intraoperative imaging. Piezoelectric transducers are responsible for generating these incredibly high-frequency waves by transforming electrical signals into vibrations in mechanical components. Pulses of sound undergo absorption, dispersion, or reflection as they pass through different types of tissues, each having its own unique acoustic impedance. A two-dimensional model of the tissue is created when the transducer picks up the returning echoes and measures how long it takes for them to return. Images can be rectangular, fan-shaped, or pie-shaped depending on the design and orientation of the transducer array (21). This gives you a lot of versatility when imaging different anatomical regions. Accurate imaging at different depths is essential in neurosurgery, which impacts the choice of transducers. Standard probes that run at 5–10 MHz can image structures in the middle of the brain because they can resolve features with a pixel resolution of 500–1,100 μm at depths ranging from 2 to 8 cm. Nevertheless, transducers that operate at up to 25 MHz provide better resolution (100–600 μm) at shallower depths (2–4 cm) for surface structures or sensitive regions that demand fine detail. Because higher frequencies are more easily attenuated and scattered within denser tissue, this resolution vs. penetration trade-off is crucial (22). The tumor's location, the surrounding anatomy, and the surgeon's unique imaging requirements determine the choice of frequency, probe type, and scanning approach. The reflected signal's amplitude is affected by the tissues' acoustic impedance gradients, which in turn affect the echogenicity, or brightness, of structures in 2D US imaging. The sulci, falx cerebri, choroid plexus, and walls of blood vessels are some examples of areas with sharp changes in acoustic impedance that seem hyperechoic (bright) because of the powerful reflections they experience. On the other hand, spaces filled with cerebrospinal fluid (CSF), ventricles, cysts, and other acoustically homogeneous areas seem dark, or hypoechoic. The echotexture of the brain parenchyma is consistent, with the exception of white matter, which appears somewhat less echoic than gray matter. Because of their greater mass density, tumors stand out visibly from the normal brain tissue and frequently appear as hyperechoic (23). Nevertheless, the diagnostic difficulty and significance of operator competence in picture interpretation are brought to light by the fact that persistent edema can resemble the echogenicity of tumors (126). 2D US has a few drawbacks despite its benefits, which include ease of use, quick picture collecting, and low cost. Problems arise, for example, when dealing with complicated anatomical structures or lesions that go beyond a single imaging plane, as this method relies on planar imaging. Chronic edema and tumors are two examples of sick and normal tissues that can look quite similar, which can lead to misunderstandings (24). Because precise probe handling and interpretation abilities are required for reliable imaging, the system's reliance on the operator's knowledge further creates unpredictability. However, 2D US is a useful tool for neurosurgery due to its low cost and ease of use; this is especially true in situations where more sophisticated imaging techniques, such as intraoperative MRI, would not be an option. The capacity of 2D US to adjust to changes that occur during surgery, such as the movement of the brain due to the removal of CSF fluid or a tumor mass, is one of its distinctive strengths. In contrast to static data provided by preoperative imaging modalities, 2D US allows surgeons to make real-time adjustments to their tactics with dynamic updates (25). As an example, if the surrounding tissues collapse or edema patterns change after excision, the tumor's echogenicity may shift. According to Asensio et al. (26), 2D US increases the chances of attaining gross total resection by continuously delivering real-time feedback, which helps with the exact identification of residual tumor tissue. Ultrasound technology is on the cusp of overcoming some of 2D US's shortcomings. Volumetric reconstructions, when combined with 3D photography, can reveal hidden details in intricate structures. Furthermore, new opportunities for distinguishing tumor tissues from adjacent abnormalities, such edema, have emerged with the advent of contrast-enhanced ultrasonography and elastography. These advancements, along with better transducer design and computational image processing, are making ultrasonic systems for neurosurgery more reliable, usable, and precise. For instance, according to Moiyadi et al. (27), contrast-enhanced US has demonstrated potential in revealing tumor vascularity, which can help differentiate between non-neoplastic and neoplastic tissues. Finally, because to its simplicity, low cost, and high effectiveness, 2D US is still an essential tool for intraoperative imaging. Its adaptability to surgical dynamics and real-time imaging capabilities make it indispensable for directing neurosurgery treatments, notwithstanding certain limits. Combining conventional 2D US with cutting-edge imaging methods will increase its diagnostic and therapeutic use in neuro-oncology as technology develops further.

#### 2.2.2 Three-dimensional ultrasound

**Figure 2:**
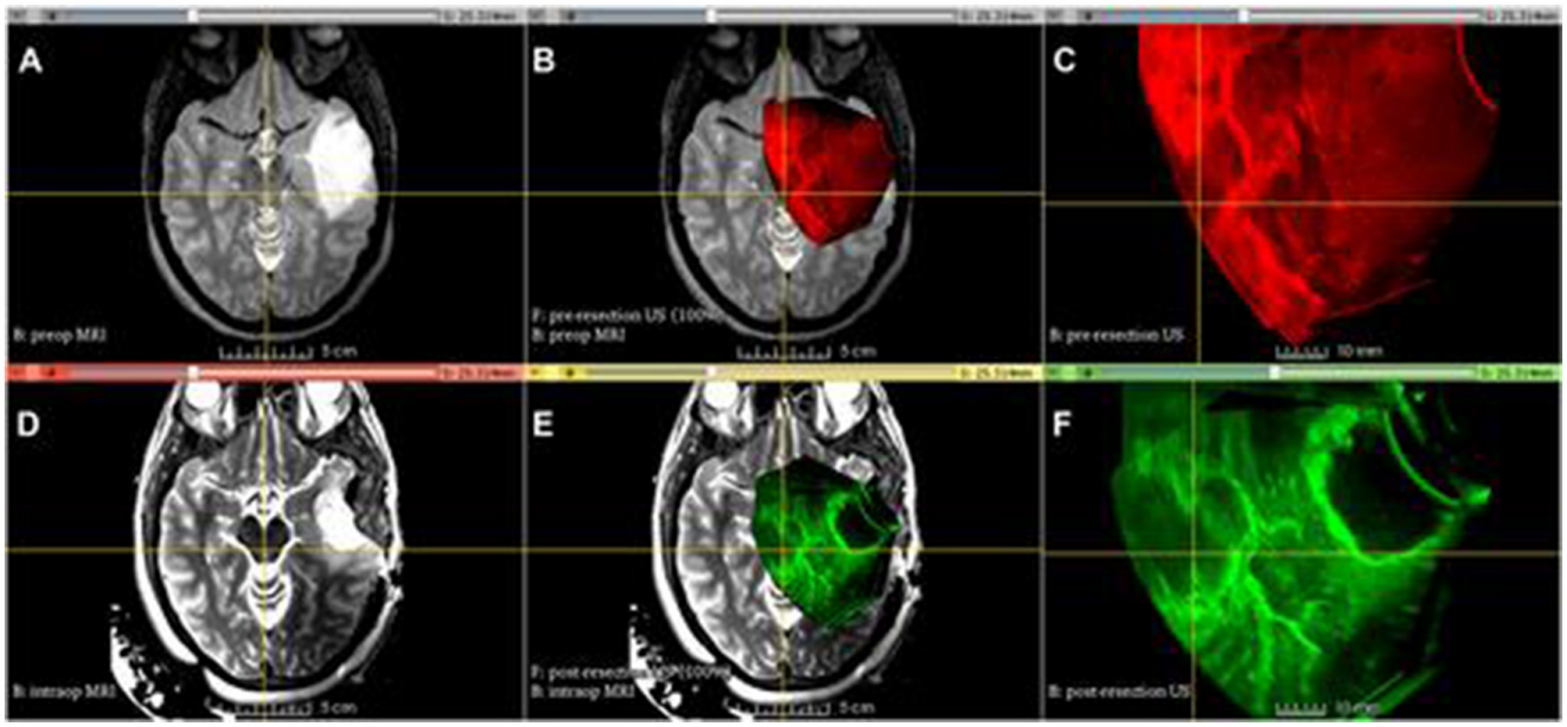
Advanced Multimodality Image Guided Operating (AMIGO) Suite at Brigham and Women's Hospital utilized tracked 3D US imaging and MRI during glioma resection. The following images show different images acquired during the procedure: **(A)** T2-fluid-attenuated inversion recovery (FLAIR) MRI before the operation, **(B)** 3D ultrasound before the durotomy, **(C)** 3D ultrasound before the durotomy, **(D)** 3D ultrasound before the durotomy, **(E)** 3D ultrasound during the operation, **(F)** 3D ultrasound after the initial tumor removal (29).

With its revolutionary ability to improve vision and accuracy during tumor resections, three-dimensional ultrasonic surgery (3DUS) has quickly become a go-to intraoperative imaging tool in neurosurgery and beyond. It offers real-time volumetric imaging with unmatched resolution and is applicable to a wide range of tumor types, including gliomas, cavernomas, medullary lesions, tumors near the skull base, and arteriovenous malformations (9, 28) (Figure 2).

Comprehensive spatial reconstructions are generated by 3DUS by combining a coronal plane with typical two-dimensional (2D) imaging. This allows for the precise determination of tumor size, volume, and spatial orientation. Research has demonstrated that these skills enhance surgical results and reduce risks associated with difficult procedures, including vascular injury and persistent tumor tissue. There is a lot of evidence that 3DUS is useful in glioma surgery. As an illustration, 74% of patients had their health-related quality of life preserved during 3DUS-guided resections of low-grade gliomas (LGG) performed under general anesthesia, according to Bø et al. (127). Reaching gross total resection (GTR) in about 74% of cases, the extent of resection (EOR) was similar to those achieved with other modern neurosurgical techniques (30). The accuracy of 3DUS in estimating the area of resection was reported to be over 80% in a study of 162 brain tumor patients, including high-grade gliomas (HGG), LGG, and other diseases. The study demonstrated the versatility of the modality by showing how it safely contributed to procedural safety and precision while being sensitive in detecting tumor boundaries and for minimally invasive trajectories. The capacity of 3DUS to adapt in real-time to changes that occur during surgery, such as brain shift, because of things like tumor removal and cerebrospinal fluid (CSF) drainage, is one of its most notable features. Conventional navigation systems that depend on preoperative imaging frequently make navigational mistakes since they don't take these modifications into consideration (31). On the other hand, 3DUS offers real-time imaging updates, so surgeons always have accurate anatomical data to work with. The use of 3D ultrasound in awake procedures for eloquent LGGs was shown by Šteno et al. (32) to greatly increase the amount of tissue that could be removed, leading to an impressive 86.79% GTR rate when compared to traditional neuronavigation methods. The fact that this method was effective and safe in high-stakes surgeries without increasing the occurrence of long-term neurological impairments is crucial. The use of contrast-enhanced ultrasound (3D-CEUS), which uses microbubble contrast agents to increase the visibility of vascular systems and tumor edges, is another breakthrough linked with 3DUS. Because tumor vascularity frequently makes excision more difficult in high-grade glioma procedures, this method is very helpful in those cases. The use of 3D-CEUS increased imaging quality in more than half of the cases, as shown by Arlt et al. (33), who found that 90% of HGGs exhibit strong contrast uptake. Finding remaining tumor tissue intraoperatively and differentiating glioblastoma borders were both demonstrated by the study's authors as benefits of 3D-CEUS. Because 3D-CEUS provides imaging quality similar to postoperative MRI, it is a dependable tool for real-time resection control, and this integration has also been successful in reducing the risk of incomplete resections (34–36). The usability of 3DUS is further enhanced by the integration of quantitative measures such as standard deviation (SD) and mean pixel brightness (MPB). With these measures, tumor echogenicity, heterogeneity, and infiltration can be objectively evaluated. According to Camp et al. (37), solid tumor components are linked to high MPB values, whereas necrotic cores and infiltrative margins are related with low MPB values. However, SD values show where the tumor and its borders have different levels of cellularity, thus they can help with surgical plane design and provide real-time insights into how the tumor is behaving. Recognizing any remaining tumor tissue during resection is an important step in achieving a more thorough excision and decreasing the likelihood of recurrence; these objective assessments are very helpful for this purpose. Although 3DUS has many benefits, it does have certain drawbacks (38, 39). Especially in cases of deep-seated cancers or intricate anatomical locations, image quality is crucial. Because understanding volumetric data necessitates competence in neuroanatomy and ultrasound principles, operator competence is crucial for optimizing the modality's potential. Even though 3DUS is more accessible and cheaper than IOMR, researchers are still working to improve its resolution and find ways to incorporate it with other imaging modalities such preoperative MRI and CT. According to Unsgaard et al. (40), this combined method is anticipated to generate extensive datasets using many modalities, which will allow for even more precise surgical procedures. To sum up, 3D ultrasound is a radical step forward in intraoperative imaging; it provides volumetric insights in real time, which improves surgical accuracy and patient outcomes. It is an essential tool for contemporary neurosurgery practice due to its versatility, low cost, and flexibility to integrate into existing surgical processes. 3D ultrasonic scanning (3DUS) has the potential to significantly impact the lives of patients dealing with complicated brain tumors if technology develops further. This includes higher resolution images, analysis powered by artificial intelligence, and hybrid imaging systems. There is mounting evidence that 3DUS is effective, thus it will continue to lead the way in intraoperative imaging innovations.

#### 2.2.3 Doppler ultrasonography

Because it provides surgeons with unmatched real-time imaging of tumor vascularity, Doppler ultrasonography has become an essential tool for brain tumor resection. Doppler effect detection of frequency shifts due to ultrasonic reflection off moving particles (such as red blood cells) is the basis of the technology. Important details on the flow rate and direction of blood are revealed by this change (41). When it comes to neurosurgery, Doppler ultrasonography is useful for a few things: finding important vascular structures, gauging the blood flow to tumors, and reducing the possibility of bleeding during tumor removal. This is accomplished by providing high temporal resolution, which is essential for comprehending intraoperative vascular changes that are dynamic and cannot be captured by static imaging modalities like as MRI or CT. Doppler ultrasonography has shown clinical usefulness in neurosurgery in multiple trials. For instance, in vascular-rich tumors, this method can decrease blood loss by as much as 30% during intraoperative procedures as compared to surgeries that depend only on preoperative imaging (42). In addition, traditional imaging frequently fails to capture the vascular complexity of deep-seated or poorly delineated tumors, but this technology has shown to be quite useful in mapping these tiny veins. A more thorough examination of the various subtypes of Doppler ultrasonography uncovers the advantages and disadvantages of each. A color Doppler imaging is a popular subgenre that uses color overlays on two-dimensional grayscale ultrasound pictures to show the degree of Doppler changes. Although this technique is angle-dependent, it does provide information regarding the direction and velocity of blood flow. Vascular structures cannot be seen because flows that are perpendicular to the ultrasound waves do not cause a detectable Doppler shift. An additional artifact that can distort the size or direction of flow is aliasing, which occurs when high velocities above the transducer's Nyquist limit. This further complicates interpretation (43). By focusing on the Doppler signal's intensity instead of its frequency shift, power Doppler imaging is able to overcome some of these obstacles. The optimum method for imaging complex vascular networks within tumors, this methodology lowers noise, enhances sensitivity to small-caliber arteries, and eliminates aliasing problems. Johnson et al. (44) compared power Doppler with magnetic resonance angiography (MRA) and found that power Doppler showed smaller vessels with 25% more precision and better single-frame arterial and venous delineation. Nevertheless, power Doppler may not be as useful in all surgical settings because it does not provide directional and velocity data. To add insult to injury, power Doppler's extreme sensitivity makes it easy to see tiny, clinically insignificant arteries, which can clog the operating room and make the technique less useful in heavily vascularized tumors. Doppler ultrasonography has great benefits, but it also has its share of problems. Because acquiring and interpreting images requires a great deal of knowledge and experience, operator reliance is still a major constraint. Vascular signals can be masked and image quality diminished by motion artifacts, which can be brought about by either the patient's movement or the probe's instability. According to research, vascular signal clarity can be reduced by 15%−20% even with very small motion errors. Doppler ultrasonography may also underestimate tumor margins because, although it is excellent at temporal resolution, its spatial resolution is still lower than that of modalities like MRI and CT. In light of these restrictions, supplementary imaging methods are required to fill in the gaps in our knowledge of tumor vascularity and architecture (45). As an example, the use of Doppler ultrasonography in conjunction with preoperative magnetic resonance angiography (MRA) or computed tomography (CT) angiography (CT) might enhance surgical results by providing both static and dynamic vascular information. This combined method was determined to decrease significant vascular problems by 18% when compared to using Doppler ultrasonography alone, according to a comprehensive review of intraoperative imaging approaches (46).

One area where this technology is seeing more and more use is in the advancement of sophisticated Doppler techniques, such 3D power Doppler imaging. The volumetric perspective of tumor vascularity provided by 3D reconstructions allows surgeons to plan resections with higher precision. When it comes to small-caliber vessels, 3D power Doppler is far more sensitive than MRA. This makes it ideal for capturing arteries and veins. The gold standard, digital subtraction angiography (DSA), and 3D power Doppler were both shown to provide comparable vascular detail in 92% of instances involving highly vascular brain tumors in a comparative study (47). However, there are certain drawbacks to using 3D power Doppler due to its great sensitivity. For example, it can overestimate the size of the arterial or show tiny vessels that don't have any clinical value. To address these challenges and make the most of Doppler's clinical relevance, it is crucial to have expert interpretation and to integrate its findings with other imaging modalities. To sum up, Doppler ultrasonography is an essential tool for removing brain tumors because it provides real-time information on the tumor's blood vessels, which makes the surgery more precise and safer. Color Doppler and power Doppler are two of its many varieties that each offer something unique to meet unique therapeutic requirements. Improvements in Doppler technology, such as 3D imaging, are expanding its uses despite drawbacks such operator reliance, motion sensitivity, and poorer spatial resolution. Neurosurgeons can improve patient outcomes by gaining a full picture of tumor vascularity through the integration of Doppler ultrasonography with other imaging modalities (48–51).

#### 2.2.4 High-frequency ultrasound

The use of linear array transducers in high-frequency ultrasound (hfUS) allows for imaging with a much higher resolution than in traditional ultrasound, thanks to frequencies reaching up to 25 MHz. Because of this feature, hfUS is an effective technique for enhancing the visualization of tumor margins, especially in difficult situations such peritumoral edema or tissue alterations caused by radiation therapy (RT) (52, 53). Research has shown that hfUS can detect tumor margins up to 30% more accurately than conventional ultrasound methods, mostly because it can pick up finer details of tissue surfaces. In brain tumor resections, where obtaining maximal resection with little injury to surrounding healthy tissue depends on precisely identifying the tumor boundaries, this enhanced resolution is very helpful. The use of hfUS has shown promising results in breast cancer procedures, with improved imaging resulting in a 15%−20% reduction in positive margin rates. This suggests that it could be useful in neurosurgery settings as well. Because its transducers operate at a higher frequency, hfUS has a limited field of view, which is one of its main drawbacks. In contrast to traditional ultrasound, which may reach deeper layers of tissue, hfUS is designed to focus on superficial structures and can usually only see structures up to 1–3 cm deep (54). But new transducer designs have made it possible to directly insert smaller, more flexible probes into resection cavities, so this limitation isn't as severe as it once was. Because of this advancement, the target tissue may be approached more closely, which improves the visibility of any remaining tumor tissue that might be missed using conventional approaches. Direct intraoperative imaging is valuable, as shown in research by Miller et al. that showed a 25% improvement in residual tumor detection rates compared to standard 2D ultrasound when hfUS probes were inserted into resection cavities. However, it is somewhat inconvenient for surgeons to only be able to see tiny bits of the resection cavity at a time. They have to move the probe around a lot to see the whole operative area. Another promising area of hfUS is its capacity to visualize peritumoral edema and post-radiotherapy alterations in great detail. This is especially noteworthy because both situations are difficult for normal imaging techniques to detect (55). The changed acoustic characteristics of edematous tissues or irradiated areas make conventional ultrasonography struggle with poor image quality. When compared to these settings, hfUS is 40% more sensitive to detect small changes in tissue. Recognizing tumor remains in affected areas requires such sensitivity since even tiny errors in resection margins can have a major influence on clinical results. One possible downside of hfUS's increased sensitivity is that it can reveal microstructural features, which may or may not correspond with clinically important pathology. This may cause the surgeon to spend more time than necessary estimating the presence of remaining tumors (56, 57). There is mounting evidence that hfUS has the ability to enhance surgical results in neurosurgery, lending credence to its use despite its limitations. One study found that compared to situations where just standard ultrasound was utilized, a retrospective examination of 50 brain tumor resections employing hfUS resulted in a 15% lower rate of residual tumor as validated by postoperative MRI (58). On top of that, hfUS could be a great addition to multimodal imaging processes because of its real-time feedback capabilities, which are in line with the aims of intraoperative navigation systems. Integrating hfUS with other imaging techniques, including intraoperative MRI or 3D ultrasound, could enhance surgery field visibility by combining the best features of each technology. To conclude, high-frequency ultrasound is an exciting new development in intraoperative imaging for the removal of brain tumors, providing better resolution and more accuracy even in difficult surgical settings. Existing limitations include a narrow field of vision and the necessity to constantly move the probe. However, as technology advances and it becomes more integrated with other imaging modalities, its potential uses are bound to grow. Surgery accuracy and patient outcomes can be greatly enhanced with the use of hfUS because of its superiority over traditional ultrasound in detecting fine tissue changes and delineating tumor boundaries. Further research is necessary to perfect its application and confirm its efficacy in various healthcare environments (59–61).

#### 2.2.5 Contrast-enhanced ultrasound

Neurosurgery is seeing the rise of contrast-enhanced ultrasound (ceUS), a technique that greatly improves the detection of cancers dependent on an ardent vascular supply by providing real-time image of tissue vascularity. The method makes use of gaseous microbubble contrast agents, which reverberate in the presence of ultrasonic vibrations. These compounds are very different from contrast agents used in CT or MRI scans since they do not diffuse across interstitial tissue, stay restricted to capillaries, and do not cause any harmful side effects (62). The attractiveness of ceUS for intraoperative imaging stems from its safety profile, which offers real-time information about the durations of arterial and venous phases, peak signal intensities, and magnitudes of contrast enhancement. Surgeons can use these factors to pinpoint tumors, gauge tumor grade, anticipate vascularity, and navigate intricate vascular structures with more accuracy. Research in hepatology, for example, has demonstrated that ceUS may detect vascularized lesions with sensitivity rates higher than 90%, suggesting that it could be useful in neurosurgery as well (63, 64). New contrast agents and sonographic algorithms have improved the spatial and temporal resolution of ceUS, allowing its inclusion into neurosurgery. One advantage of ceUS over Doppler imaging is its ability to detect both high- and low-flow arteries at the same time, regardless of the angle of insonation. With this capability, a more complete image of tumor vascularity can be painted by extensive mapping of tumor microcirculation and perfusion dynamics. In instances of highly vascular brain tumors, a study conducted by Lee et al. (65), showed that ceUS reduced residual tumor rates as confirmed by postoperative MRI, and it also detected tumor boundaries with 25% more accuracy than traditional 2D ultrasound. There is some evidence that ceUS can be useful for assessing tumor-level perfusion heterogeneity, which is a proxy for cancer grade. Because of these features, ceUS is a great tool for making decisions during surgery, particularly when dealing with aggressive tumors that have complicated blood supply or complicated vascular anatomy. Although ceUS has many benefits, it is still not widely used in neurosurgery due to a number of constraints. To begin, there is occasionally a great deal of interoperator variability due to the specialized knowledge needed to optimize imaging parameters. During contrast agent delivery, surgeons must maintain a steady vision, which can restrict their maneuverability. Unfortunately, not all surgical centers have the high-tech ultrasound equipment that is needed for this technological necessity (66). Furthermore, imaging must precede the coagulation of tumor-feeding arteries due to the intraoperative nature of the contrast agent, which may cause a disruption in the surgical workflow. According to research, these changes to the workflow can add up to fifteen percent to the total operating time, which could make ceUS impractical in high-volume surgical facilities. Not only is there no set protocol or set of guidelines for the use of contrast agents in tumor excision, but there are also no FDA-approved agents for use in neurosurgery in the US, which further complicates their routine administration (67). For surgical procedures involving tumor microcirculation, ceUS offers a safer alternative to conventional imaging modalities such as CT or MRI because it does not require ionizing radiation or nephrotoxic chemicals. Its intraoperative usage, however, has complications that must be carefully considered. For instance, it is still very difficult to adapt to changing surgical settings while keeping image quality high. According to Vetrano et al. (68), intraoperative ceUS was found to have better tumor vascularity imaging than non-contrast ultrasonography. However, the study did note that there was a steep learning curve for successful use and longer setup times compared to non-contrast ultrasound. The results highlight the importance of consistent training programs and equipment in order to get the most of ceUS while minimizing its drawbacks. To sum up, contrast-enhanced ultrasonography is a game-changer in intraoperative imaging; it helps surgeons understand the microcirculation and vascularity of tumors like never before. In order to improve the accuracy of tumor resections, surgeons rely on its capacity to capture perfusion patterns in real time (69). However, there are still technical, logistical, and regulatory hurdles that prevent its full incorporation into neurosurgical workflows. Improving surgical outcomes for patients with highly vascularized brain tumors is possible with the further development of standardized techniques and the possibility for ceUS to become a staple of neurosurgical imaging. Its use and the validity of its long-term advantages in neurosurgery require additional research (68) (Table 1).

**Table 1:** Clinical applications and insights of contrast-enhanced ultrasound (CEUS) in glioma surgery.

**Cases**	**Study points**	**Methodology**	**Clinical insights**	**Conclusion**	**Key advantages**	**Challenges**	**Time**	**Reference**
87	Investigated the integration of CEUS with fluorescein sodium to improve tumor boundary and residual identification during surgery	Combined CEUS and fluorescein sodium for real-time intraoperative imaging during glioma resections	Enhanced tumor boundary identification, better differentiation from normal brain parenchyma, increased extent of resection (EOR), and reduced residuals	CEUS with fluorescein sodium is a real-time, safe, and effective method for identifying tumor boundaries and residuals, improving EOR and protecting neurological function	Real-time visualization; easy to integrate with neuronavigation; low cost	Requires additional intraoperative agents like fluorescein sodium	2024	Fang et al. (70)
64	Assessed the survival and prognosis impact of CEUS-guided glioma resections compared to those without CEUS	Retrospective study comparing survival rates and prognoses of patients undergoing glioma surgery with and without CEUS integration	CEUS highlighted residual tumors, improving the completeness of resections. Age and CEUS use were identified as key prognostic factors for surgical success	CEUS facilitates residual tumor identification and is a prognostic factor for malignant glioma surgery, improving patient outcomes	Improved survival outcomes; better prognosis for high-grade gliomas	Operator dependence; variability in tumor vascularization patterns	2024	Chen et al. (71, 72)
51	Explored the utility of routine intraoperative ultrasound (IU) and CEUS in brain tumor surgery	Compared routine IU with CEUS for intraoperative localization and outlining of brain lesions	CEUS proved more effective for hypervascular lesions, aiding in lesion localization and residual tumor identification	CEUS is more effective than routine IU for outlining hypervascular lesions and residual tumors, enhancing total lesion removal	High sensitivity for vascular lesions; dynamic intraoperative imaging	Potential for noise and artifacts in resection cavity	2022	Tao et al. (73)
49	Analyzed quantitative CEUS parameters and their relation to microvessel density (MVD) in gliomas	Quantitative CEUS imaging was performed alongside MVD assessment in different glioma grades	Quantitative parameters (e.g., perfusion rates) correlated closely with MVD, aiding in tumor grading and preoperative and intraoperative strategy refinement	CEUS parameters closely correlate with MVD, providing real-time imaging and quantitative data that aid in glioma grading and surgical strategy optimization	Offers quantitative data for tumor grading; continuous dynamic imaging	Limited availability of quantitative CEUS in all centers; requires advanced software and expertise	2019	Wang et al. (74)
25	Investigated CEUS in low-grade gliomas (LGG) to evaluate residual tumor after resection	CEUS used intraoperatively to monitor resection cavities and detect residual low-grade gliomas	Improved detection of small residuals compared to standard B-mode ultrasound, aiding in complete tumor removal in LGG cases	CEUS provides enhanced real-time visualization for residual tumor detection in low-grade glioma surgeries	High resolution for LGG; enhanced tumor cavity evaluation	Potential false positives due to artifacts or gliosis	2018	
10	Evaluated CEUS's specificity in identifying residual tumor masses during glioma surgery	CEUS imaging combined with B-mode ultrasound for glioblastoma resections	High specificity in distinguishing tumor tissue from artifacts or normal parenchyma based on vascularization patterns	CEUS accurately identifies residual tumors by assessing vascularization, crucial for maximizing glioblastoma resections	High specificity for glioblastoma; real-time decision-making tool	Less effective for distinguishing small tumor remnants surrounded by edema	2016	Prada et al. (75)
5	Provided clinical data on CEUS's versatility in neuro-oncology	Case series analyzing CEUS in routine neuro-oncological surgeries	Demonstrated dynamic imaging capabilities, eliminated anatomical distortions from neuronavigation, and provided real-time perfusion data for improved tissue differentiation and intraoperative guidance	CEUS is a safe, dynamic, real-time imaging modality that eliminates anatomical distortions, provides perfusion data, and aids in intraoperative diagnosis, tissue differentiation, and EOR quantification	Versatility in neurosurgery; eliminates reliance on static imaging	Learning curve for operators; image quality can vary with anatomical location	2016	Lekht et al. (76)
120	Evaluated CEUS's diagnostic significance in assessing brain glioma resection degree	Transmission electron microscopy (TEM) alongside CEUS was used to analyze residual tumor rates	CEUS improved glioma resection rates, offering high sensitivity and specificity in detecting residual tumor tissue when compared to TEM	CEUS demonstrated high sensitivity and specificity for tumor excision evaluation and improved resection rates for gliomas when combined with TEM	Enhanced sensitivity for residuals; compatible with histological analysis (TEM)	Medium concordance rates between CEUS and histopathology in certain cases	2015	Yu et al. (77)
100	Examined CEUS's ability to improve visualization of tumor vascularity in glioblastoma resections	CEUS combined with T1 MRI to analyze the extent of resection and tumor vascularity in real time	CEUS provided superior visualization of vascular structures compared to T1 MRI, improving intraoperative decision-making	CEUS enhances real-time understanding of tumor vascularity, contributing to safer and more effective glioblastoma resections	Better vascular imaging; complementary to MRI	Limited data on its standalone accuracy without adjunctive imaging	2014	Zhou et al. (78)
69	Characterized cerebral gliomas using CEUS	CEUS performed intraoperatively to differentiate between glioma types	Showed potential in distinguishing malignant from benign gliomas dynamically and cost-effectively, facilitating surgical strategies and intraoperative decision-making	CEUS is fast, safe, dynamic, and cost-effective for differentiating malignant from benign gliomas and refining surgical strategies	Cost-effective; reliable differentiation of tumor grades	May require specialized training to interpret vascularization patterns	2014	Prada et al. (79)

## 3 Ultrasound elastography

One imaging method that can map tissues' elastic characteristics by studying their stress response is ultrasound elastography (UE). This technique measures strain, which is the relative deformation of tissue under applied stress, and provides information about stiffness, a characteristic that is frequently associated with pathological alterations. Since its 1991 description, ultrasound elastography has been useful in detecting breast tumors, fibrosis in the liver, and prostate cancer. Elastography has been investigated as a possible intraoperative imaging adjunct for brain tumor surgery, with the aim of better distinguishing the tumor from normal brain tissue. Elasticography has the potential to improve tumor excision accuracy by capitalizing on mechanical contrasts between edematous, softer tissue and pathological, harder parts. Uncertainties in histological correlation and substantial imaging noise, however, continue to restrict its utility (80). 2D or 3D methods of intraoperative elastography are available, with strain maps produced by active compression (e.g., vibrography of the axial probe) or passive detection of pulsations in the artery wall. While passive methods rely on physiological pulsations to decrease the need for external forces, they introduce variability, while active compression approaches provide greater control over the mechanical input, which could lead to improved consistency. In contrast to 2D approaches, 3D ultrasonic elastography has the potential to better visualize variations in tissue stiffness, which could help in spatially delineating tumor edges (81). The significant amounts of noise that are intrinsic to elastography data gathering continue to be a hurdle, even with current advancements. Its usefulness in differentiating tumor from healthy tissue may be diminished if noise obscures delicate tissue distinctions, which complicates interpretation. The dependability of ultrasonography elastography in brain tumor procedures is greatly impacted by the operator. If you want reliable findings every time, you need a lot of experience with image acquisition and interpretation. While there is encouraging evidence between elastography and histology, the results have been variable among research. As an example, elastography has the potential to distinguish high-grade gliomas from normal tissue with a sensitivity of up to 80%, according to some findings (82). While some have emphasized its uniqueness, others have pointed out the variety in strain patterns and how they overlap among tumor types. The necessity for standardized methods and training to enhance operator proficiency and interobserver agreement is highlighted by this disparity (83).

The extremely short acquisition and processing times of elastography make it a promising tool for providing surgeons with immediate feedback on their work. Elastography is a safer and more economical alternative to traditional imaging technologies because it does not include ionizing radiation or contrast chemicals. But it can't capture fine details because to its low resolution and motion artifacts (84). When dealing with tumors that are either poorly defined or deeply embedded in the body, these restrictions become even more apparent since the mechanical characteristics of the surrounding tissue can obscure even the most minute tumor-related alterations. The use of elastography in conjunction with other intraoperative imaging modalities, such as intraoperative ultrasonography or magnetic resonance imaging (MRI), shows great promise despite these limitations. Some of the present limitations of ultrasound elastography may be overcome by future developments, such as the incorporation of AI for automated interpretation. Strain maps could be made more accurate with the help of machine learning methods that can improve correlation with histology and decrease noise (85). Moreover, as 3D elastography methods advance, they may enable thorough observation of tumor stiffness fluctuations, which would be helpful for surgical navigation. The capacity of elastography to offer non-invasive, real-time evaluations of tissue stiffness makes it a useful tool for enhancing the accuracy and security of brain tumor resections, even if it has not yet attained broad use in neurosurgery. To realize its full potential and determine its place in clinical practice, additional research and technological improvement are required (86).

### 3.1 Integration with neuronavigation systems

A major step forward in neurosurgery imaging has been the incorporation of neuronavigation systems with intraoperative ultrasound (US), which permits high-resolution, real-time imaging of tumors and their environs. To maximize the extent of resection (EOR) and improve patient outcomes, it is crucial to use a US modality that can effectively localize and differentiate the tumor. Different US modalities have different strengths and shortcomings in this regard. The use of two-dimensional ultrasonic scanning (2D US) to measure extraoperative radiation (EOR) and tumor volume correlates well with preoperative magnetic resonance imaging (MRI), especially in cases of gliomas and metastatic tumors (87). 2D US offers more accurate results in first resection cases than in recurrent surgeries, according to studies. This is probably because the echogenicity is lower in the former case, thanks to the previous radiation or surgery. The complementing role of US with MRI is further demonstrated by its ability to detect tumor bulk beyond the margins evident on gadolinium-enhanced and non-enhanced T1-weighted MRI and to differentiate tumors from edema in T2-weighted imaging. Ultrasound (US) accomplishes diagnostic yield rates that are comparable to computed tomography (CT) for stereotactic brain biopsies, further demonstrating its value in neurosurgery (88). By creating volumetric models of tumors, three-dimensional ultrasound (3D US) allows for more accurate delineation of histological boundaries and better visibility overall. The study found that compared to biopsy-confirmed pathology, 3D ultrasound had a 74% concordance rate for low-grade astrocytomas, an 83% rate for anaplastic astrocytomas, a 77% rate for glioblastomas, and a 100% rate for metastases. Based on these findings, 3D US can outperform T1-weighted MRI and compete with T2-weighted MRI when it comes to defining tumor borders. Brain changes caused by tumor removal and edema reduce 3D US's sensitivity and specificity, which can compromise the technology's accuracy during surgery. For instance, glioblastoma detection sensitivity dropped from 95% before resection to 26% after (89). However, when combined with 5-aminolevulinic acid (5-ALA) during fluorescence-guided surgery, 3D US has shown an exceptional ability to improve EOR in non-enhancing gliomas, despite these limitations. Intraoperative Doppler ultrasonic scanning (US) allows for the real-time localization of important blood arteries, which brings a vascular component to tumor imaging. The precision of 2D color Doppler imaging in glioma resections is limited because to brain movements, although it has been utilized to map vascular structures onto preoperative imaging. To get around this, 3D power Doppler imaging was created so that surgeons can avoid using preoperative imaging altogether while navigating around vascular systems (90). Tracked 2D power Doppler identified vascular structures after hemangioblastoma removal that would not have been observed with conventional 2D ultrasound. These innovations boost the surgeon's self-assurance while dealing with complicated anatomy, make resection safer, and lessen the likelihood of vascular injury. By enhancing US with contrast, tumor vascularity and perfusion dynamics can be better understood, significantly expanding US's value. Microbubbles used in ceUS imaging are restricted to capillaries rather than the interstitium, making them ideal for a more direct evaluation of blood flow than contrast chemicals used in CT or MRI (91). While gliomas and lymphomas show less pronounced patterns on ceUS, meningiomas, hemangioblastomas, and metastases have substantial contrast enhancement. Among seventy-one patients with brain tumors, ceUS revealed that glioblastomas were diverse, exhibiting quick vascular phases and significant enhancement, in contrast to low-grade gliomas, which showed slower perfusion. Although false positives are likely in situations of recurrent glioma with prior irradiation due to gliosis-related abnormalities, ceUS has demonstrated 62% sensitivity and 93% specificity in detecting residual tumor. Results like these highlight how important it is to improve and standardize ceUS in neurosurgery procedures. By mapping tissue stiffness, a new modality called ultrasound elastography (UE) could be incorporated into neuronavigation systems (92). Ultrasound (UE) can distinguish tumors from normal parenchyma by assessing strain caused by axial probe vibrations or arterial pulsations. The use of strain imaging for dissection plane prediction was supported by a study on brain tumor resections, which showed mechanical differences between the tumor and adjacent tissue. Noise, operator reliance, and weak association with histology are some of the issues that the technology encounters. Additional study is required to establish the reliability of UE across tumor types and grades, and the integration with neuronavigation is still in its infancy. In comparison to more traditional US techniques, high-frequency ultrasound (hfUS) provides far better resolution when it comes to detecting tumors. Compared to 2D US (24% sensitivity) and gadolinium-enhanced T1-weighted intraoperative MRI (55% specificity), hfUS attained a sensitivity of 76% for tumor detection in glioblastoma procedures. On the other hand, compared to 2D US (96%) and MRI (74%), its specificity was inferior at 58% (92). With less medically produced artifacts, hfUS showed sensitivity for low-grade gliomas that was comparable to T2/FLAIR MRI (79 vs. 83%). Using hfUS and confirmed by MRI after surgery, one study found a gross total resection rate of 95.5%. More work is needed to perfect its use, though, because superficial resolution limits and insufficient scanning could cause remaining tumors to be missed. Neuronavigation systems rely on dynamic imaging modalities such as US to overcome the difficulty of brain shift, which is the relocation of brain structures after surgery. Preoperative imaging can be rendered inaccurate due to brain displacement, necessitating updates in real-time to ensure accuracy. Although there are certain technological challenges due to different picture qualities and artifacts between US and MRI, registering the two allows for updates that reflect intraoperative changes. Research utilizing both rigid and non-rigid registration methods has shown discrepancies of 3–5 mm, with inaccuracies caused by probe calibration and fiducial misalignment. To improve registration accuracy, methods like Bayesian frameworks and hyperechoic structural matching are being investigated (93). Problems with registering anatomical landmarks are also encountered by neuronavigation systems that incorporate the US. Though it has demonstrated better accuracy for intracranial characteristics, postdurotomy 3D US registration still has problems with cortical alignment impacted by brain shift. Updates during tumor excision are provided by US, however surgical procedures can introduce artifacts. The necessity for improved algorithms to handle intraoperative variability has been brought to light by studies that have documented 3.2 mm misalignment in subcortical locations. Regardless of these issues, US is a great tool for adjusting preoperative planning to intraoperative realities since it can give real-time information. Multimodal imaging, new registration methods, and AI-driven image analysis are some of the future possibilities for US-integrated neuronavigation. Improved histopathological correlation and noise reduction are two ways in which AI algorithms could improve US interpretation. Systems like ceUS and 3D Doppler have the potential to provide a thorough picture of tumor margins, vascular anatomy, and tissue perfusion when used together (94). In order to hone these technologies and determine their function in enhancing surgical results, additional research is required. Finally, neuronavigation systems that use ultrasound modalities improve the accuracy of neurosurgery procedures by supplementing preoperative planning with real-time, dynamic imaging. Ongoing developments have great potential to enhance the utility of US during difficult tumor resections, despite the fact that there are still challenges with registration accuracy, artifact reduction, and uniformity.

### 3.2 Emerging experimental technologies in intraoperative ultrasound for glioma surgery

**Figure 3:**
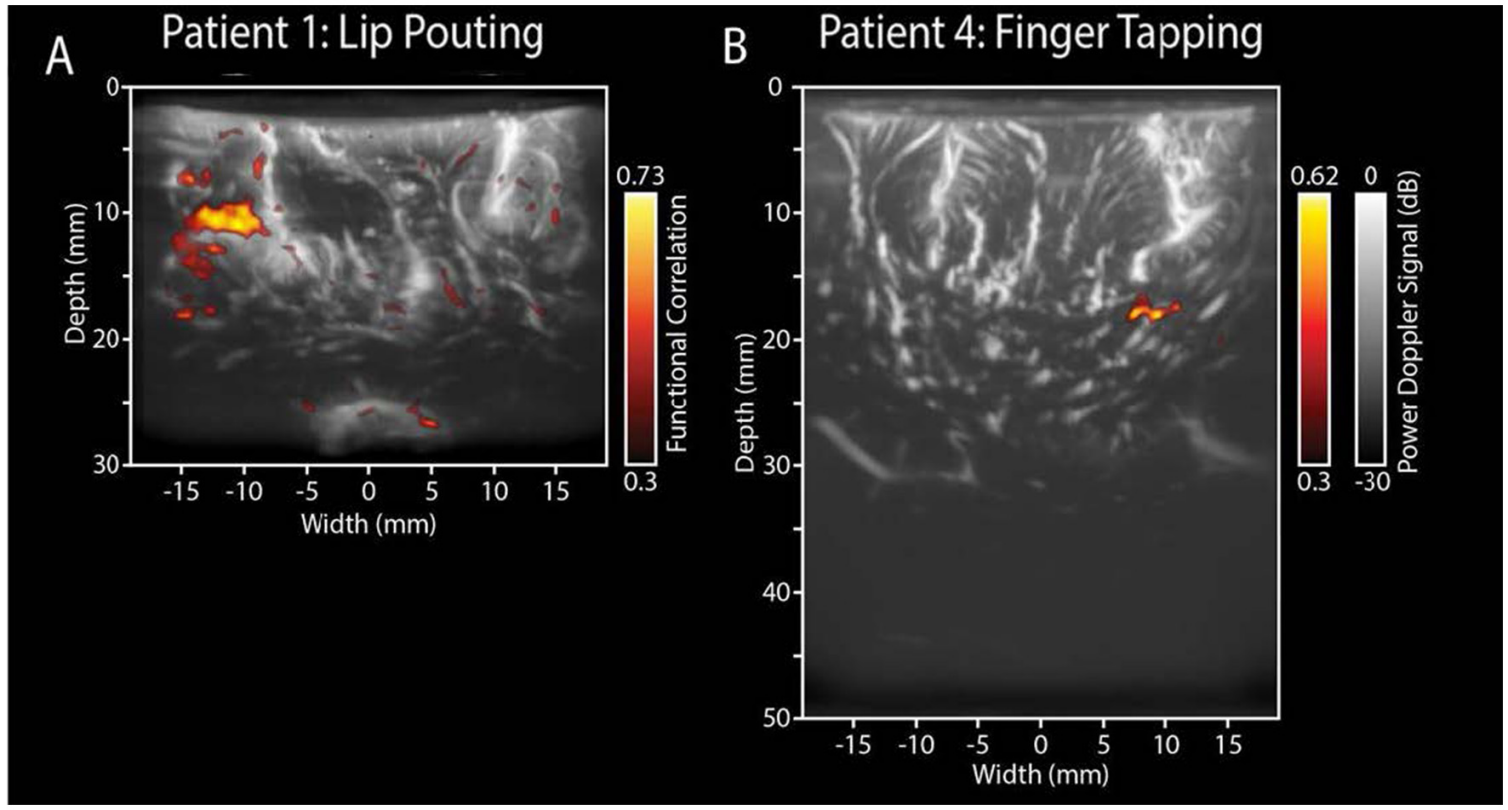
Functional ultrasound results of two functional motor tasks. Real-time fUS displays functionally active voxels during tasks. **(A)** ntraoperative electrocortical stimulation mapping (ESM) confirmed the tumor's proximity to the primary motor cortex. During surgery, the patient was shown a 60-s video task involving lip-pouting, alternating between three 8-s task blocks and three 10-s rest blocks, with a 6-s baseline. Functional imaging revealed brain activity near the tumor cavity in response to the task within a 3.8 cm × 3.0 cm area. **(B)** The recorded functional response closely follows the task pattern (yellow line), indicating activation in relevant motor areas. In contrast, non-functional regions do not exhibit this task-related activity (white line).

Functional ultrasound (FUS) is rapidly gaining clinical relevance in brain tumor surgery due to its superior ability to visualize task-evoked brain activity intraoperatively. In a pioneering clinical study, Imbault et al. (95) Demonstrated the feasibility of intraoperative FUS in detecting localized blood volume changes during motor tasks with an exceptional spatiotemporal resolution of 250 μm and 1 ms. The authors used ultrafast Doppler imaging during surgical procedures to measure dynamic hemodynamic changes related to functional activity. This method captured activity in deep cortical areas, a capability often lacking in fMRI or electrocorticography. The clinical relevance is substantial: real-time mapping of functional areas during tumor resection reduces the risk of postoperative deficits while enabling more aggressive tumor removal. Building on this, Soloukey et al. (96) FUS was applied intraoperatively to patients performing predefined motor tasks. In Figure 3 of their work, two exemplary cases demonstrate how FUS reveals motor cortex activation near tumor margins. In one case, Figure 3A shows cortical hemodynamic responses evoked by a lip-pouting task, with activation precisely localized near a recurrent glioblastoma cavity. In another, Figure 3B captures deeper activation during a finger-tapping task, demonstrating FUS's depth penetration up to 5 cm. Beyond motor mapping, FUS has also been applied to language processing tasks. The FUS correlation maps revealed different spatial patterns for overt and covert speech, with eloquent zones confirmed by both preoperative fMRI and intraoperative electrocortical stimulation.

Artificial intelligence (AI) is revolutionizing intraoperative ultrasound by enhancing image interpretation through deep learning models, particularly Convolutional Neural Networks (CNNs). These models are capable of segmenting tumor boundaries, fusing intraoperative ultrasound with preoperative MRI, and detecting tumor margins with high specificity and speed.

Del Bene et al. (97) advanced ultrasound imaging techniques—such as Doppler, elastography, and contrast-enhanced ultrasound in 28 glioma surgeries, highlighting how grayscale interpretation could be augmented through computational approaches. Although this study did not use CNNs directly, it laid the groundwork for quantitative image features such as echogenicity profiles and edge sharpness that later informed AI training sets. In parallel, Moiraghi et al. (98) Evaluated a navigated intraoperative ultrasound (N-ioUS) platform combined with neuronavigation and discussed the integration of AI-based tools in real-time. In a prospective cohort of high-grade glioma (HGG) patients (*n* = 42), N-ioUS significantly improved the extent of resection (EOR) compared to conventional 2D ultrasound. Tumor boundaries were better visualized intraoperatively, and when fused with preoperative MRI through AI-assisted registration algorithms, the updated image compensations for brain shift were highly accurate. These outcomes directly impacted survival by increasing complete resection rates without increasing neurological morbidity.

The application of 4D ultrasound, which captures dynamic volumetric data over time, has been explored for tracking tumor resection in real-time. In clinical practice, 4D iUS facilitates continuous assessment of the resection cavity and is particularly valuable in regions where tumors infiltrate deep or non-cohesive brain structures. Šteno et al. (8) conducted a multi-institutional analysis of intraoperative ultrasound in glioma surgeries and emphasized that quantitative grayscale metrics such as Mean Pixel Brightness (MPB) and Standard Deviation (SD) can be used to differentiate tumor from surrounding tissues. They noted that gliomas typically show lower homogeneity (higher SD) and increased hyperechogenicity (higher MPB) compared to peritumoral edema or healthy cortex.

Theranostic contrast agents—particularly nanobubbles designed for ultrasound activation represent an exciting frontier in neurosurgical oncology. While clinical applications are still in early phases, studies have highlighted their dual capabilities: acting as contrast enhancers and drug delivery vehicles. Moiraghi et al. (98) Noted preliminary efforts in using microbubbles functionalized with ligands targeting glioma-specific markers such as EGFR or VEGF. Once injected intravenously, these microbubbles accumulate in tumor vasculature and can be acoustically triggered to release their contents ranging from doxorubicin to siRNA.

Although large-scale human trials are pending, small-scale translational studies have shown enhanced visualization of tumor perfusion, and in murine glioma models, nanobubble-mediated delivery of chemotherapeutics has significantly improved local cytotoxicity while sparing surrounding tissue. In surgery, these agents could provide real-time fluorescence or acoustic feedback upon contact with residual tumor, guiding the final resection stages more precisely than visual inspection alone.

## 4 Innovations in ultrasound technology for glioma surgery

### 4.1 Navigable ultrasound systems

Due to improvements in intraoperative imaging and an increase in the extent of resection (EOR), glioma surgery has been revolutionized by advances in ultrasound technology. A major step forward in this area is the development of navigable ultrasound systems (NUS), which integrate preoperative data with advanced navigation systems and combine the real-time advantages of conventional intraoperative ultrasound (IOUS). With this setup, neurosurgeons have a reliable, uninterrupted path to follow when they operate on the brain's intricate architecture. Patients with non-eloquent high-grade gliomas had a considerable improvement in EOR and neurological outcomes when navigable ultrasonography was combined with preoperative MRI (99), according to a study by Moiraghi et al. (98) By combining real-time NUS with conventional neuronavigation alone, tumor volumes larger than 1 cm3 might be better detected, proving that real-time NUS supports more thorough tumor resections while lowering the chance of residual malignant tissue. Not only do navigable ultrasound systems enhance EOR, but they have other benefits as well. In a study of 210 cases, Shetty et al. (100) showed that NUS might find unexpected tumor remnants during surgery, which could influence surgical decisions for the better and encourage more aggressive resection when it was safe to do so. Because of intraoperative brain shifts, which occur naturally during surgery as a result of variables including cerebrospinal fluid release and gravity, this study demonstrated that NUS have the ability to detect extra tumor masses that traditional neuronavigation would miss. To improve resection control and lessen the effect of brain shift, NUS keep the surgeon up-to-date in real-time with input that allows them to alter the surgical trajectory and bring the intraoperative landscape into line with preoperative imaging. The capacity to integrate with other imaging and functional mapping techniques is a key feature of navigable ultrasound technology. To improve the accuracy of navigation around key structures and the precision of anatomical orientation, Rueckriegel et al. (101) investigated the use of probabilistic fiber tracking in conjunction with navigable ultrasound within the FMRIB software library. This synergy allowed for more assured intraoperative adjustments, which in turn allowed for safer resections in expressive brain regions and allowed for three-dimensional estimate of brain shifts. While NUS do improve resection accuracy, the study found that it really shines when paired with sophisticated neuronavigation algorithms that take into consideration actual anatomical changes in real time and integrate into the workflow without causing any major disruptions. Navigable ultrasound systems have many advantages, but they also have certain drawbacks. When it comes to deep-seated lesions in particular, studies like Patil et al. (102) have shown that 3D ultrasound-guided devices have drawbacks, such as lengthier operating times and increased technical demands. In many cases, the benefits of these approaches much exceed the drawbacks, particularly when dealing with complicated or deeply situated gliomas. Although navigable 3D ultrasound offers greater precision and resolution for deep-seated malignancies, free-hand 2D ultrasound is still useful for more superficial lesions. Biopsies and resections, where accuracy is of the utmost importance, benefit greatly from NUS's three-dimensional visualization and capacity to compensate for intraoperative changes, which contribute to improved diagnostic yields and less risky results. In order to make NUS even more reliable and useful for glioma surgery, researchers will likely concentrate their efforts on enhancing picture quality, reducing reliance on operators, and incorporating AI for automated interpretation in the near future (103) (Table 2).

**Table 2:** Summary of clinical studies on navigated ultrasound (NUS) in glioma surgery.

**Cases**	**Study points**	**Methodology**	**Clinical insights**	**Conclusion**	**Key advantages**	**Challenges**	**Impact on patient outcomes**	**Technological requirements**	**Time**	**Reference**
210	Investigated the use of NUS as an intraoperative adjunct for resection control in diffuse gliomas where EOR is critical	NUS integrated with iUS for real-time imaging and navigation guidance	Enhanced detection of unanticipated tumor residues and improved resection rates when combined with functional mapping techniques	NUS is a useful adjunct for glioma resection, positively influencing surgical outcomes, but resection depends on tumor resectability and relationship to eloquent areas	Real-time imaging, navigation integration, and improved EOR detection rates	Requires combination with functional mapping for resection near eloquent areas	Higher EOR leading to increased progression-free survival; reduced neurological deficits when used with mapping	Integration with neuronavigation platforms; high-resolution NUS probes; experienced surgical teams	2021	Shetty et al. (100)
31	Evaluated the impact of combining NUS with preoperative MRI for maximizing EOR in glioma surgery compared to standard methods	NUS combined with conventional neuronavigation using preoperative MRI for non-eloquent high-grade gliomas	Improved neurological outcomes and greater EOR, with better detection of residual tumor volume >1 cm3 compared to standard neuronavigation	NUS-based real-time imaging promoted better EOR and outcomes, especially for non-eloquent high-grade gliomas	Enhanced EOR and neurological outcomes; reliable residual tumor detection	Limited impact on eloquent gliomas; residual volumes < 1 cm3 may still pose challenges	Increased functional outcomes in non-eloquent gliomas; higher accuracy in residual tumor detection	Seamless data fusion with preoperative MRI; availability of dynamic NUS-compatible neuronavigation systems	2020	Moiraghi et al. (98)
125	Compared free-hand 2DUS and navigated 3DUS for ultrasound-guided biopsies of supratentorial lesions	Free-hand 2DUS for superficial lesions; navigated 3DUS for deep-seated lesions	NUS improved accuracy for biopsies of deep-seated supratentorial lesions despite longer operative times and slightly higher complication rates	NUS offers greater utility for deep-seated lesion biopsies, while free-hand 2DUS is more suitable for superficial lesions	Effective for deep-seated lesions; provides high accuracy in challenging locations	Longer operative times; higher postoperative complication rates compared to free-hand 2DUS	Improved diagnostic yield in challenging lesions; safer biopsy procedures for deep-seated tumors	Need for 3DUS-enabled probes and neuronavigation software capable of handling deep-lesion imaging	2019	Patil et al. (103)
11	Assessed the feasibility of integrating probabilistic fiber tracking and NUS into intraoperative neuronavigation	NUS combined with FMRIB software library-based probabilistic fiber tracking for anatomic orientation during glioma resection	Enhanced three-dimensional estimation of brain shift during surgery, improving the reliability of neuronavigation and intraoperative decision-making	Integration of probabilistic fiber tracking and NUS facilitated preoperative and intraoperative workflows, enabling better anatomical orientation during glioma resections	Improved accuracy of neuronavigation; accounts for brain shift in real-time	Technical complexity of integrating probabilistic fiber tracking with intraoperative workflow	Higher precision in brain shift compensation; improved safety in eloquent area resections	Advanced neuronavigation systems with probabilistic tracking software and dynamic brain shift compensation algorithms	2016	Rueckriegel et al. (101)

### 4.2 Functional ultrasound applications

By providing unparalleled spatiotemporal resolution for measuring brain activity and vascular dynamics, functional ultrasonography (FUS) has become a game-changing imaging technique in glioma surgery. The infiltrative development pattern of gliomas is what makes them so dangerous; they frequently invade highly functional regions of the brain that are involved in speech, motor skills, and sensory processing (104). It is crucial to preserve these areas during surgical resections in order to reduce postoperative problems and improve patient quality of life, but this infiltration makes the process more complicated. Capturing neural activity within deep brain regions or distinguishing minor vascular dynamics can be challenging with traditional functional neuroimaging techniques like functional magnetic resonance imaging (fMRI). The majority of hemodynamic responses that indicate brain activity occur in small arteries, and standard 2D ultrasonography has trouble detecting this flow. A reliable proxy for brain activity through neurovascular coupling mechanisms, FUS overcomes these limitations by using ultrafast plane-wave imaging to track transient changes in cerebral blood volume (CBV). By precisely localizing functional areas, FUS allows for maximal tumor excision while protecting essential brain connections, thanks to its spatial resolution of up to 250 μm and temporal precision of as fine as 1 ms (105).

Several important investigations have shown that FUS can map functional areas during surgery in real time. Accurate localization of functional areas of the cerebral cortex was demonstrated by Mace et al. (106) using FUS to capture CBV changes associated with task-evoked neuronal activation. The capacity to accurately distinguish tumor tissue from healthy functional areas is crucial for surgeons to make decisions during surgery. Research by Imbault et al. (95) demonstrates the usefulness of FUS in various surgical settings by identifying and mapping cortical activation during task-evoked responses in awake and anesthetized patients. Since FUS can show hemodynamic responses in deeper sulci and subcortical locations, it is very useful for treating gliomas that invade beautiful but physically complicated areas, and its use is not limited to cortical areas. All of these new developments highlight how FUS might improve surgical results by providing high-resolution feedback in real-time, closing the gap between structural and functional imaging.

New developments have broadened the use of FUS in glioma surgeries. In this study, Soloukey et al. (96) demonstrated that FUS can differentiate between healthy and tumor-related vascular features and record task-evoked cortical responses, highlighting its potential use in awake brain surgery. Gliomas benefit greatly from this dual capability because, in order to achieve maximal safe excision, it is necessary to identify infiltrative tumor margins from surrounding vasculature. Quantitative ultrasound velocimetry (vUS) has also expanded functional imaging's capabilities. After developing vUS to detect axial and transverse blood flow velocities, Tang et al. used *in vivo* observations and computational simulations to confirm the accuracy of their method. With its ability to withstand high-frequency noise and acoustic attenuation, vUS outperforms traditional FUS tools, guaranteeing reliable operation in demanding surgical settings. Its promise for functional imaging in glioma surgery on humans is demonstrated by its capacity to track changes in blood flow as a function of brain activation, like whisker stimulation in animal models. A major step forward in neuroimaging has been the expansion of focal ultrasound imaging from 2D to 3D and eventually 4D. By utilizing tomographic techniques, Demené et al. (107) expanded FUS capabilities to whole-brain 4D vascular neuroimaging, allowing for full visualization of brain hemodynamics. Although this method has only been tested on rats so far, it could have far-reaching consequences for glioma surgeries on humans. More accurate demarcation of functional regions might be possible with real-time 3D imaging of brain vasculature during tumor removal, decreasing the likelihood of postoperative impairments. Quantitative blood flow evaluation and contrast-free imaging are both on the rise, which bodes well for the future of FUS in healthcare. A further selling point of FUS is the possibility of integrating it with neuronavigation systems and AI-based analysis to provide surgeons with dynamic and adaptable guidance. The integration of FUS into everyday neurosurgical workflows is nevertheless not without its hurdles, despite these developments. Unfortunately, most neurosurgeons still rely on static preoperative imaging methods that can't adjust to changes that occur during surgery, such brain shift. An alternative that shows promise is FUS, which can give real-time anatomical and functional data. The difficulty in understanding hemodynamic responses and the requirement for specific training to operate ultrafast imaging equipment are two examples of the technological limitations that prevent its widespread use. However, these obstacles should be overcome with the further development of high-resolution probes, automated image analysis, and user-friendly interfaces, rendering FUS an essential component of glioma surgery. Substantial shifts will occur in neurosurgical practice as the technology develops and plays an ever-larger role in maintaining patient functionality while optimizing tumor excision (108, 109).

## 5 Advanced therapeutic applications of ultrasound

Recent advancements have expanded the role of focused ultrasound (FUS) in glioblastoma (GBM) therapy beyond imaging, positioning it as a versatile platform for non-invasive tumor ablation, targeted drug delivery, immunotherapy enhancement, sonodynamic therapy. Among these, high-intensity focused ultrasound (HIFU) has demonstrated the capacity to thermally ablate tumor tissue through localized hyperthermia exceeding 55 °C, leading to coagulative necrosis, protein denaturation, and structural disruption of glioma cells (110, 111). Early human trials, including MR-guided FUS following craniectomy, showed promising results with histological confirmation of thermocoagulation, though complications such as inadvertent damage to adjacent healthy tissue due to low targeting precision were noted (112). More recent innovations, such as intraparenchymal HIFU catheters, have enabled direct delivery of heat within brain tissue, bypassing skull attenuation and enhancing accuracy, particularly in preclinical GBM models (113). Additionally, FUS-induced hyperthermia has synergized effectively with radiation therapy (RT), increasing radiosensitivity by impairing DNA repair pathways, suppressing stemness-related signaling (e.g., Akt), and potentiating immune-mediated responses (114, 115).

A transformative function of FUS lies in its ability to transiently and locally disrupt the blood-brain barrier (BBB), a major obstacle to effective chemotherapeutic and biologic delivery in gliomas. The combination of FUS with intravenously injected microbubbles initiates oscillation-driven mechanical effects (sonoporation) that reversibly open tight junctions between endothelial cells, enabling the penetration of systemically administered agents such as temozolomide, doxorubicin, and monoclonal antibodies (116). Moreover, this technique facilitates targeted nanoparticle delivery, enhancing intratumoral accumulation of imaging probes, chemotherapeutics, and gene vectors. Studies have shown improved therapeutic efficacy when drugs are encapsulated in ultrasound-sensitive nanocarriers, which release their payload in response to sonication, thereby reducing off-target toxicity (117, 118). MRI-guided FUS protocols have further refined BBB modulation by allowing real-time thermal and spatial monitoring to ensure safety and precision (119).

FUS is also emerging as a key enabler of immunotherapeutic strategies. By opening the BBB, FUS enhances delivery of immune checkpoint inhibitors (ICIs) which are otherwise poorly distributed within the CNS (120, 121). It also improves infiltration and activity of engineered immune cells, addressing their previously limited efficacy in GBM due to poor tissue penetration and antigenic heterogeneity. FUS-induced mechanical effects stimulate tumor antigen release and activate antigen-presenting cells (APCs), including dendritic cells, effectively transforming the tumor site into an *in-situ* vaccine and amplifying systemic antitumor immunity (122).

Finally, FUS-mediated radiosensitization represents another promising direction. FUS has shown potential to assist glioma surgery by opening the BBB and facilitating targeted drug delivery intraoperatively, though clinical use remains experimental (123). Furthermore, FUS-induced hyperthermia contributes to vascular remodeling, enhanced perfusion, and greater accumulation of radiosensitizers, such as nitroimidazole derivatives and 5-ALA, all of which collectively heighten GBM's responsiveness to irradiation (123). Integration of real-time MR-guidance ensures precise thermal targeting, reducing collateral damage to surrounding brain tissue. Despite encouraging preclinical outcomes, translation into clinical application still requires standardization of sonication parameters and identification of patient-specific biomarkers to optimize therapeutic outcomes (124, 125).

In summary, focused ultrasound is establishing itself not only as an intraoperative imaging adjunct but also as a multifaceted therapeutic tool in glioblastoma treatment. Its capacity for non-invasive ablation, precision drug delivery, and immune modulation offers a platform for combination strategies that may significantly improve patient outcomes when integrated into future multimodal treatment protocols.

## 6 Future perspectives and concluding remarks

Imaging modalities, particularly intraoperative ultrasound (IOUS) and its integration with cutting-edge technology like neuronavigation systems, will play a pivotal role in the development of future brain tumor surgical procedures. Radical resection is the main determinant affecting patient survival, and IOUS has already shown to be an invaluable tool in attaining this. With IOUS, surgeons can receive real-time input, unlike with preoperative imaging modalities like MRI and CT, and make dynamic adjustments as needed. Anatomical distortion caused by brain displacement during craniotomy is a major restriction of neuronavigation, but this skill solves it. Neuronavigation and IOUS work together to improve tumor margin identification, which in turn allows for more accurate resections and better patient outcomes. Reliability as an intraoperative imaging technique has been shown by studies showing a high correlation between IOUS use and postoperative MRI results in gliomas and metastatic brain cancers.

Some of the weaknesses in existing systems will be remedied by the introduction of cutting-edge technology in the United States. For example, compared to traditional 2D US, high-frequency ultrasound (hfUS) has better resolution, allowing it to pick up on tiny tumor remains. Although hfUS can sometimes match the sensitivity and specificity of MRI, aberrations and noise from the resection cavity make its intraoperative application difficult. Innovative acoustic coupling fluids and enhanced probe designs are two examples of the efforts that are showing promise in overcoming these obstacles. In addition, neuronavigation devices that incorporate real-time 3D US imaging could improve visualization of tumor margins and resection cavities, leading to a further reduction in the likelihood of leaving residual tumor tissue.

CEUS, or contrast-enhanced ultrasonography, is yet another tool that can improve imaging during surgery. Differentiating tumor tissue from normal parenchyma and surrounding edema is much easier with the help of CEUS, which provides comprehensive vascular maps of tumors. Research using glioblastomas and meningiomas found that CEUS was very good at pinpointing tumor borders and leftover tissue. The CEUS microbubbles provide a safer alternative to MRI and CT contrast agents by remaining inside the vasculature, eliminating the risks of radiation exposure and nephrotoxicity. The diagnostic and therapeutic potential of CEUS could be further enhanced by future improvements in the technology, such as the creation of contrast chemicals that are specifically designed for certain types of tumors. Elastography is an additional technique that shows promise; it distinguishes between tumors and normal brain regions by using the mechanical characteristics of the tissue. Elastography has demonstrated promise in mapping tumor stiffness and predicting dissection planes during surgery, however it is still in its early phases of clinical application. Before elastography may be widely used, however, problems like operator dependence, noise levels, and limited correlation with histology need to be solved. Surgeons could be given more precise and repeatable data during resections if elastography is combined with AI-driven interpretation tools, which would alleviate these limitations. Focused ultrasound (FUS) will certainly play a larger role in the imaging and therapeutic applications of brain tumor surgery in the future, alongside these technological developments. Problems caused by skull anatomy and inefficiency in peripheral brain regions are the focus of current FUS efforts. The range of uses for FUS is anticipated to be expanded by innovations such as patient-specific ultrasound arrays, non-thermal ablation procedures, and enhanced ultrasound focusing. Potentially inaccessible brain cancers could be amenable to FUS-based targeted drug delivery and blood-brain barrier disruption in future clinical studies. Treatment of intracranial metastatic disorders could be transformed, for example, by FUS-mediated administration of viral constructs or nanoparticle medication carriers. An other game-changing prospect is the use of AI and ML into intraoperative imaging systems. Artificial intelligence algorithms can improve tissue differentiation, decrease noise, and compensate for artifacts, all of which improve image interpretation. When integrated with multimodal imaging systems, AI has the potential to provide accuracy and dependability that exceed what humans are currently capable of. For instance, by combining information from imaging modalities such as MRI, CT, and IOUS, surgeons would have a more precise picture of the operative field, which would allow for more precise and safer resections. At long last, we must resolve the practical and ethical concerns that arise from using this cutting-edge technology. It will be more important to guarantee equal access to the benefits of these systems as they evolve. Care inequalities may emerge as a result of the prohibitive expense of neuronavigation platforms, high-tech ultrasound machines, and related educational initiatives. The only way to make these technologies accessible to everyone is for scientists, medics, businesses, and governments to work together on a worldwide scale. To confirm the usefulness, safety, and cost-efficiency of new instruments, extensive multidisciplinary research and clinical trials are required. Finally, developments in ultrasonography and its incorporation with neuronavigation and therapeutic technologies are setting the stage for future breakthroughs in brain tumor surgery. Improving surgical precision, maximizing tumor resection, and improving patient survival while reducing complications are all possible outcomes of these improvements. But getting there will need overcoming existing obstacles, encouraging collaboration across disciplines, and making sure these innovative treatments are available to all patients. More promising results in some of medicine's most difficult cases may be on the horizon as neurosurgery undergoes a sea change over the next two decades as a result of relentless study and technical advancements.
